# The transformation-mainstreaming conundrum: Making sense of tensions in adaptation practice

**DOI:** 10.1007/s13280-025-02271-0

**Published:** 2025-11-21

**Authors:** Roger Street, Michael Dunlop, Seona Meharg, Russell Gorddard, Yiheyis Maru, Minh N. Nguyen, Deborah O’Connell, Rachel Williams, Russell M. Wise, Mark Stafford Smith

**Affiliations:** 1 University of Oxford, Oxford, OX13QY UK; 2 CSIRO Environment, Black Mountain, GPO Box 1700, Canberra, ACT 2601 Australia; 3 Green Templeton College, University of Oxford, 43 Woodstock Road, Oxford, OX2 6HG UK; 4 Australian National University, Canberra, ACT 2601 Australia

**Keywords:** Climate adaptation, Governance, Mainstreaming climate action, Transformational change

## Abstract

As the scale of climate change impacts become apparent, organisations globally are seeking to adapt. They face dual imperatives of transformation—going beyond business-as-usual to embrace disruptive changes to their decision-making processes—and mainstreaming—enacting adaptation initiatives with minimal change to existing capabilities and structures. In practice, these important imperatives can conflict, leading to the emergence of multiple tensions in developing and implementing adaptation initiatives, potentially paralysing action or leading to one imperative dominating. We call this the Transformation-Mainstreaming Conundrum (TMC) and suggest that both imperatives can (and must be) pursued simultaneously in practice. This perspective identifies recognisable tensions that can arise when seeking to address both imperatives and suggest steps towards responding to the underlying issues these tensions reveal. The TMC needs to be recognised, and approaches to navigating its tensions must be addressed explicitly in both scholarship and practice, to re-energise the urgency of scaling up adaptation efforts.

## Introduction

Climate adaptation scholarship and practices have evolved and matured since the Third Assessment Report (IPCC 2001, 2022). These developments reflect the widespread and urgent need to identify adaptation options that can be effective and readily implemented. These must also respond to the emerging magnitude of climate impacts that require long-term forward planning for major and pervasive action. There is a long-standing and complicated knowledge-action gap (Castree 2021) reflecting the many drivers that affect policy-makers’ decisions as well as perceived gaps in the science. Practice indicates an increasing need for reflexivity, focusing on learning from adaptation efforts, where there is a growing understanding of the fundamental shifts in behaviour and decisions required (Preston et al. 2015; Colloff et al. 2017; O'Connell et al. 2020; Swart et al. 2023). The result has been calls for adaptation to be both *transformative* [interventions that change the fundamental attributes of a social–ecological system in anticipation of the scale and pace of emerging climate change impacts (Global Commission on Adaptation 2019; World Bank 2019; IPCC 2022)] and *mainstreamed* [interventions that are integrated into existing policy-making, budgeting, implementation and monitoring processes at national, sector and subnational levels, to close the existing and emerging adaptation gaps (Rogers et al. 2024a; UNFCCC 2015; European Commission 2019; Global Commission on Adaptation 2019)].

In this perspective article, we identify a series of tensions that arise in practice between these two imperatives. We propose that these tensions need to be navigated directly, maintaining a simultaneous commitment to both imperatives so that adaptation is both effective and implementable. We have observed these tensions while conducting participatory adaptation research with governments, NGOs, utilities and businesses around the world. In this perspective, we draw specifically on examples from Australia, Vietnam, Papua New Guinea and the UK. However, we find that they are supported by the experiences of others in the global literature. They emerge particularly where research has pointed to the need to disrupt the status quo (to embrace transformative change) and yet to integrate and implement those changes within existing policy and institutions (to mainstream). We suggest that there is an underlying phenomenon at play that needs to be recognised and addressed explicitly in both scholarship and practice, in order to re-energise the urgency of scaling up adaptation efforts. We identify this phenomenon as the transformation-mainstreaming conundrum (TMC).

To this end, the paper outlines the two imperatives in more detail and hence defines the TMC; then, we develop a preliminary list of key tensions that it can cause, before identifying some approaches that help to navigate these tensions and ending with a call for more attention to be paid to the phenomenon.

## Transformation and mainstreaming imperatives

### The transformation imperative

The magnitude of existing and projected climate impacts and the sensitivity of many natural, built and social systems call for fundamental changes in the relationships between society and the physical and natural systems people depend on. These include significant changes in the flows of services and benefits and how these are sustainably managed (Kates et al. 2012; Colloff et al. 2017; West et al. 2019; Colloff et al. 2021). Such actions will involve configuring new system relationships at different scales and within different domains (e.g. Chan et al. 2019). The associated negotiations will involve conflicts and trade-offs, are likely to be disruptive socially, institutionally and politically, and may require different forms of governance (Morrison 2016; van Kerkhoff et al. 2019; West et al. 2019; Colloff et al. 2021; Muiderman et al. 2022).

Identifying and implementing transformative adaptation (Lonsdale 2015; Fedele et al. 2019; Filho et al. 2022; Shi and Moser 2021) often requires questioning or opposing the status quo, going beyond marginal changes that seek to resist or accommodate small levels of change with minimal disruption to the goals and processes of work and life (O’Brien 2012; Nelson 2013; Dow et al. 2013; Pelling et al. 2014; Kasdan et al. 2020; Wilson et al. 2020). Transformative adaptation measures often require significant changes in behaviour, social values, political contexts, processes and mechanisms, including how risks and vulnerabilities are assessed, and decisions are made (Tengö and Andersson 2022). Required changes are likely to meet barriers to uptake and implementation and stand apart from incremental changes by involving “entirely new practices … complete changes in mindset, major shifts in perceptions or values”, and being “widespread”, “rapid” and “challeng[ing] hard limits” (Kates et al. 2012; Berrang-Ford et al. 2021). In addition, they will not necessarily be the natural endpoint of repeated short-term incremental reactive adaptation measures unless those steps are part of an intentional transformative framework (Wise et al. 2014). Despite these challenges, the imperative of transformation is an essential rallying point and boundary object for addressing global climate change (Carter 2017; Fedele et al. 2019; IPCC 2021).

### The need for mainstreaming

The imperative to mainstream adaptation has two related implications. First, climate adaptation considerations need to be readily incorporated into assessments, planning processes, policies and implementation practices, without additional projects or resources. Second, climate impacts need to be considered and implemented in all sectors and across many, if not most, functional roles, rather than having the responsibility for adaptation planning and implementation placed on environment departments or specialists (Adger et al. 2005; Dovers 2009; Scoville-Simonds et al. 2020). Mainstreaming is seen to: enable greater consistency and integration with other existing priorities (Butler et al. 2022); provide opportunities for leveraging and scaling actions (Pal et al. 2019); help identify whose mandate, responsibility and expertise the required actions belong to and potentially bridge the gaps between national and local-level (Lebel et al. 2012) and sectoral-focused planning and implementation.

The mainstreaming of adaptation can be an opportunity for taking action that leverages existing processes, resulting in minimal disruption to those who need to adapt and to the social, institutional, regulatory and political environments in which they work. In these cases, mainstreaming can enable responsible decision-makers to start addressing adaptation within the scope of their existing jobs and their mandated or legislated roles and responsibilities. However, the constraints on these roles and responsibilities often create a barrier to engaging with the requirement for transformative levels of adaptation (Rogers et al. 2024a).

### The transformation-mainstreaming conundrum

Many adaptation initiatives seek to address both imperatives, taking deliberate actions to ensure that they are both *effective* (i.e. address the magnitude of the problem, often requiring transformation) and *implementable* (i.e. mainstreaming, by making use of existing institutions and structures). However, with many such initiatives across a multitude of contexts, we have observed the emergence of systematic tensions arising from incompatibilities between the two imperatives. By labelling the TMC, we seek to draw attention to the fact that doing both is at once highly desirable and very often fundamentally challenging. For example, individuals within a government agency may seek to mainstream approaches to local planning that aim to transform regional development trajectories and encourage radical retreat from low-lying coastal towns (as canvassed in regional plans supported by South Australia's state government; Siebentritt (2014). This is clearly an important issue, yet it may raise political tensions in the agency more generally and with their stakeholders, and these tensions can undermine action. In such cases, there are discussions about both transformation and mainstreaming taking place within the same (or related) organisations, and the tensions represent legitimate differences of perspective that need to be navigated rather than avoided.

From a theoretical point of view, conflicts can arise for multiple reasons. In some cases, organisations are not internally homogeneous, so some actors may seek substantial change while others manage for stability; but also, transformation often proceeds as the outcome of multiple smaller, coordinated changes (Colloff et al. 2021). In other cases, one organisation may be mainstreaming processes that aim to encourage “client organisations” to transform (e.g. provincial governments requiring transformative adaptation at local government levels or funding programmes aiming to drive transformation in recipient organisations). Any of these models of implementing adaptation can create situations in which a part of the overall adaptation process is transformative, while other parts need to be mainstreamed into business-as-usual, triggering the TMC. As the next section shows, this results in a set of potential tensions that can undermine action; these need explicit recognition.

## Tensions and conflicts and their implications

The TMC can be experienced as one or multiple tensions in the design and implementation of an adaptation initiative. These tensions can be experienced within a single organisation, where multiple elements of its usual practice are inconsistent with some of the transformative changes anticipated. Where an organisation (or a part of it) seeks to carry out transformative adaptation itself or through others, but the need to do this must be embedded in business-as-usual practices, the TMC occurs when elements of those practices act as barriers or are inconsistent with the processes of seeking transformative change. Tensions are likely to be more extreme in situations where governance and implementation occur across a system of organisations, such as layers of government, supply/value chains or networks of cooperating entities. For example, a homeowner wishing to re-build a more fire-resilient house may need to deal with a lack of experienced designers, builders lacking knowledge about how to build the design efficiently, unavailability of materials, restrictive national building standards and municipal regulations, and insurance companies unsure about how to price their change in risk. Some individuals may be willing to navigate these issues and bear the cost, but mainstreaming the option to build such housing easily across many local council areas would require substantial change in multiple organisations.

Overarching tensions arise where the decision-making processes default to business-as-usual when, in fact, these processes need to tolerate (or change to) accept novel considerations that support transformative adaptation. Even though individuals may be invested with good intent to drive transformative adaptation, the organisation as a whole may see adaptation as a problem to be solved within current processes. This normally limits adaptation to adopting perspectives of marginal change, addressing local and deterministic challenges and using existing skills and leadership styles within an existing theory of change. Tensions arise when the transformative adaptation needed requires change at multiple levels of both context and outcome. This often involves emergent and evolving system change (supported by adaptive learning in the face of uncertainty) and demands different objectives and forms of leadership (O’Connell et al. 2018; Crosweller and Tschakert 2021; Crosweller 2022; Rogers et al. 2024a). Our observations of these issues playing out practically in organisations lead us to the following list of tensions (Table 1).

**Table 1:** Seven tensions that emerge from experience and the literature as a result of the Transformation-Mainstreaming Conundrum (TMC), elaborated and exemplified in the text

Tension
1. *Capacity to rethink objectives*: commitment to existing objectives *vs* accepting the need to reassess objectives
2. *View of system dynamics*: cyclic and stationary *vs* complex and emergent
3. *Locus for adaptation action*: adapting management *vs* adapting societal decision-making systems
4. *Framing of adaptation*: narrow and finite *vs* broad, ongoing and adaptive
5. *Response to uncertainty*: aim to reduce *vs* accept and work with
6. *Risk assessment norms*: familiar isolated hazards and controls *vs* novel and interconnected hazards, and systemic and evolving control processes
7. *Leadership style*: conventional and hierarchical *vs* coordinated, emergent and distributed

A key tension between mainstreaming and transformation is the **capacity to rethink objectives**. Objectives are often well-established and currently desirable, and the prospect of change may be seen as a threat by those who are invested in them personally or professionally. Current objectives are often embedded implicitly in existing systems and structures with no agreed processes for changing them. Change may need to happen at higher decision-making levels, and people often have limited agency to change the objectives guiding their work even if they want to. Thus, “capacity to re-think” encompasses both *mechanisms* and *willingness* to question current objectives. For example, conservation policy and projects in Australia have often been reluctant to address the reality that past objectives, such as “maintain historic species distributions” within a protected area or jurisdiction, are unachievable in the face of shifting ecosystems. The scope for seriously exploring new climate-ready objectives (Dunlop et al. 2013) with conservation practitioners and stakeholders is limited by current objectives being pervasive in the practice of managers, assessment, societal conservation narratives, teaching, research, research funding, policy and legislation. Similar inflexible objectives have been found in the emergency management sector (O’Connell et al. 2018) and have been noted widely in discussing implementation deficits (Dupuis and Knoepfel 2013; Milhorance et al. 2022), and in “the tension between engendering transformation and returning back to normal” (Schipper et al. 2021).

The willingness to acknowledge and review objectives is also associated with how existing organisations perceive their **system dynamics**. Incumbent organisations have established their mainstream processes based on an essentially stationary and predictable (if complicated) system, where, once established, proven procedures can be expected to continue to work in future. However, most systems are complex and emergent, with continually evolving dynamics (Snowden and Rancati 2021), especially in the face of rapid climate change. This tension regularly plays out in projects with limited time, scope and understanding of the context, such as international development projects where what are perceived to be straightforward cause-and-effect interventions have the potential to become ineffective or maladaptive as the messy and interconnected nature of the challenges they seek to address becomes apparent (Scoville-Simonds et al. 2020; Snowden and Rancati 2021; Butler et al. 2022; Butler et al. 2024). Similarly, many policy appraisal frameworks do not address the full system dynamics of societal and technological change sufficiently (Blythe et al. 2018), failing to consider the need to reform decision-making or re-evaluate societal preferences. For example, the Australian disaster management policy system sought a better complex systems understanding of disasters (O’Connell et al. 2018; O'Connell et al. 2020) but struggled to operationalise these concepts in the sector’s implementing agencies that were focused on short-term emergency response actions.

A related tension found across a wide range of projects concerns the perceived **locus for adaptation action**. Organisations tend to frame action around proximal impacts and responses within their own purview, where their agency is high. However, responding effectively to the levels of change being experienced may require a focus on decision-making processes and the performance of the system as a whole, and hence require engagement with other actors, as well as coordinated action across the system and the evolution of the societal decision context (Gorddard et al. 2016). There is often a focus on individual assets, organisations, impacts or projects and a lack of willingness and ability to engage in systemic scale thinking, analysis and reform that might prioritise changing structures or social norms before undertaking the adaptation process (Rogers et al. 2024a). For example, emergency and disaster management agencies in Australia (Australian Government 2018; O’Connell et al. 2018; O'Connell et al. 2020) focus on hazards that are within their agency, such as hazard reduction burning, whereas they really need to deal with vulnerabilities to climate extremes that are outside of their sphere of control, such as planning regulations, or resilience investment cases for local government assets (Tieman et al. 2022; Meharg et al. 2024; Xenarios et al. 2024). In these examples, the source of the tension is a limited locus, often due to a lack of relevant cross-scale or cross-domain institutional considerations; this limits systemic responses. Similarly, a regional adaptation group in South Australia realised that a cross-sectoral and cross-jurisdictional adaptation effort was needed following a succession of drought, flood and bushfire events (Siebentritt et al. 2014; Siebentritt 2014). However, they initially struggled to gain support from the State Government, for which regional-scale adaptation was novel at the time and challenged by the need to involve multiple local councils and sectors where the State Government had limited agency. Conservation agencies often focus on how to adapt their management of current priority threatened species and communities, rather than consider what new processes are needed to understand and encode protection of those aspects of nature (such as diversity, even if novel) that might be valued by society once ecosystems transform (Dunlop et al. 2013). Similar effects are often described in the literature where policies and practices need to be challenged (Geels 2011), power dynamics managed (Scoville-Simonds et al. 2020) and fragmented policy agendas and budget lines addressed (Peris and Bosch 2020; Milhorance et al. 2022). However, this literature does not necessarily recognise that these are inevitable tensions to be navigated.

This tension leads to the related issue of the **framing of adaptation.** Here, institutions not only tend to focus on their own locus of control, but also they see planning and funding adaptation as a once-off or periodic problem-solving process rather than a response to be designed as continuous learning and improvement processes (Tieman et al. 2022; Wise et al. 2024). This often leads to a siloed framing of the adaptation required (e.g. a biophysical framing of ecological issues or an engineering framing of bridge heights for flooding). This tension also results when the framing does not include the social and institutional context that shapes decision-making and, hence, fails to incorporate engagement and governance processes. To a large extent, this limited framing relates to the organisational scope of climate risk and adaptation assessments, drivers for the associated planning (such as national reporting requirements and corporate social responsibility) and lack of capacity (Rogers et al. 2024b; Wise et al. 2024). An inability or lack of willingness to share data and decision-making processes across organisations contributes to this tension, especially in the context of system-wide adaptation for which transformative changes may be required. This limited perspective can lead to an incomplete understanding of the risks, and identification of adaptation options that, in turn, lead to conflicts and missed opportunities for synergistic action.

Many administrative and political processes favour once-off initiatives like an adaptation plan or a specific funding programme against well-defined outcomes. Yet, such plans and programmes often need to encourage adaptive experimentation from those they support to achieve transformative adaptation. Thus, Peris and Bosch (2020) note aspects of this tension in efforts to incorporate sustainability measures into urban planning in València, Spain, seeing formal decision-making procedures set against reflexivity and social learning, as well as standardised project formats impeding open processes of searching and experimentation.

These issues are, in turn, related to tensions around the **response to uncertainty** where decision-makers are apprehensive about uncertainties, and hence seek to reduce them to enable confident decision-making and to mitigate risks. Types of uncertainties include physical uncertainties (state, dynamics and effectiveness of management) and social and institutional uncertainties and ambiguities (willingness to accept risk of action, roles and responsibilities, changing societal preferences and coordination across the system of interest). For example, policy institutions usually prefer to (imagine that they can) deal with known levels of change and simple cause-and-effect interventions (Wise et al. 2024) when the future is often irreducibly uncertain and complex social-ecological systems rarely respond simply to “command-and-control” actions. This has consequences for funding programmes that are held accountable for fixed outcomes rather than for good processes; as a result, experimentation and adaptive management are under-resourced. The tension is visible in many projects where clients, funders and stakeholders desire a reduction in uncertainty to better inform their decision-making rather than being willing to work within uncertainty and ambiguity (Butler et al. 2022, 2024). This impetus towards perceived certainty occurs despite the availability of approaches to dealing with uncertainty, such as robust decision-making and adaptation pathways (Lempert et al. 2010; Cradock-Henry et al. 2023). Such intolerance for uncertainty can be deeply embedded, as in an urban water project in Vietnam, where the country’s centralised, top-down governance system led to fear of loss of control or of making mistakes. These fears hindered innovation and resulted in missed opportunities for change; in this case, increasing recognition of the consequences has resulted in progress towards distributing autonomy and encouraging innovations in decision-making (Butler et al. 2024; Wise et al. 2024).

Associated with the response to uncertainty are tensions related to **risk assessment norms**, which include prevailing concepts, narratives, frameworks, resource allocations and capabilities. These are often out of step with what is needed to consider future climate risks, which are unprecedented and poorly characterised (O’Connell et al. 2018; Wise et al. 2024). For example, standard approaches struggle to handle unknown likelihoods and consequences, uncertainty is often interpreted as low likelihood, and systemic risks are excluded (e.g. Mercure et al. (2021) with respect to guiding decisions about the low-carbon and climate resilience transition). The tension arises where mainstream risk assessment processes are narrow and reductionist, targeting individual known hazards. Yet, the entities they seek to influence need to consider systemic and integrated risks, including compound and cascading hazards (Miro 2022; Romanach et al. 2024). In a stationary context, risk is often framed as the risk of change from the current state, whereas in the context of a transformation, risk may need to be framed as loss due to the failure to adapt, and risk mitigation framed as efforts to steer transformative change. This tension arose working with stakeholders across various projects which attempted to better understand the complex, systemic risks and disaster as an intersection of hazard, exposure, vulnerability and response (Australian Government 2019). In the emergency response sector in Australia, the federal ministry wanted a better approach to assessing systemic risk but implementing this was in strong tension to the view of individual hazards held by organisations on the ground in the sector.

This, along with the other tensions, highlights the significance of the **leadership style** tension, between conventional and hierarchical structures compared to an alternative coordinated but distributed and emergent style (e.g. (Cletzer and Kaufman 2018)). The latter allows greater flexibility and learning, consistent with the challenges associated with transformative adaptation, and it enables others to lead where appropriate in a complex system (Crosweller 2022; Rogers et al. 2024a). This tension can be due to apprehension, a lack of willingness or a lack of capability for distributed, coordinated leadership where many people play different roles (O'Connell 2018; Meharg 2023). It can also result in leadership that inhibits transformative adaptation. These leadership style tensions were observed as a particular challenge in the emergency sector, where there are often clear and defined governance and decision-making roles that are vital for providing clarity in emergencies, but which may limit innovation and experimentation in the face of long-term change (O’Connell et al. 2018; Wise et al. 2024).

Transformation-mainstreaming tensions typically result from multiple, systematically linked processes that need addressing in combination to mitigate the underlying causes of the TMC. For example, hierarchical leadership can result from institutional structures, rules and norms, and suppress experimentation and learning. Siloed processes and governance can then exacerbate these processes, as the TMC is often experienced as multiple context-specific tensions over time and place and, it may not be identified as multiple instances of a single phenomenon. In our experience, the existence of the underlying TMC is often not recognised or accepted. This leads to individual reactions to each observed tension, rather than diagnosis as indicators of the need to systematically and purposefully balance the competing imperatives and drive desirable change. As a result, the impacts of the TMC are approached in an unfocused manner and are exacerbated by the prevailing knowledge and competencies in both leaders and staff, as well as institutional arrangements, rather than seeking innovation. Some of these factors are not readily (or even, in some contexts, desirably) changed.

## Responses in practice

**Fig. 1:**
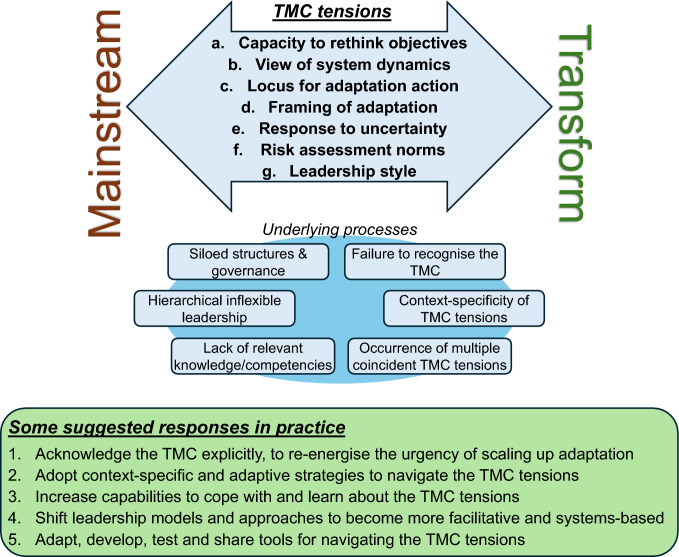
The seven tensions between the mainstreaming and transformative imperatives that are identified arising from the Transformation-Mainstreaming Conundrum (TMC), noting some processes that contribute to experiencing the tensions and some responses practitioners can use to design constructive and action-oriented approaches to navigate the tensions

The tensions resulting from the TMC are often interpreted as barriers to adaptation and can lead to frustration and confusion, as well as stalled implementation and descoping. To avoid tensions, an adaptation initiative may have its transformative intent weakened (e.g. by using a shorter timeframe or by focusing on “on-ground” actions at the cost of addressing policy objectives or organisational processes) or it may have its mainstreaming intent reduced (e.g. by restricting stakeholder engagement). Yet, the urgency of scaling up adaptation actions and considering transformative impacts is increasingly obvious, and the occurrence of tensions should be regarded as a potential indicator that the correct issues are being addressed rather than as barriers. There is a growing set of examples of adaptation initiatives that have implemented specific features to navigate tensions arising from the TMC. For example, a regional climate planning initiative in South Australia (Siebentritt et al. 2014; Siebentritt 2014) had an atypical leadership team of regional leaders, consultants, researchers and a senior bureaucrat that was able to drive a participatory process focused on building legitimacy with an otherwise sceptical local constituency, including persuading senior government figures to avoid framing the resulting programme as a government initiative. The analysis of the tensions and examples like this suggest some critical considerations for confronting the TMC and enabling greater scaling of effective climate adaptation (Fig. 1).

First, adaptation practitioners must **acknowledge the TMC explicitly, to re-energise the urgency of scaling up adaptation** efforts. Naming the tensions and normalising discussion about them will enable all those involved to understand that these are usually an outcome of mismatched system processes rather than deliberate avoidance, or some perceived impossibility, of the adaptation challenge. Schipper et al. (2021) pointed out that navigating the turbulent nature of transformation requires space for inclusive and even contested politics, which do not need to be aggravated by misunderstanding the causes of potential conflict. Recognition can also open up helpful discussion about the nature and scope of adaptation possible, including the realities of implementation. This can enable the process to hold true to both transformation and mainstreaming imperatives while avoiding lock-ins and short-term actions that strengthen the status quo (Conti et al. 2021; Buzási and Csizovszky 2023; Garrett et al. 2024; Morrison et al. 2024).

Second, it is important to **adopt context-specific and evolving strategies to navigate the TMC tensions**. There is no simple, universal playbook here, and navigating the tensions does not mean eliminating them (Smith and Lewis 2011). However, some general strategic steps can be articulated—recognise the TMC, diagnose what tensions arise in the specific context at hand (starting from Table 1, but acknowledging that it is incomplete), seek out organisational commitment to actively consider and address these tensions rather than ignore them, and aim to tackle them with innovation and organisational learning, whatever this may mean in context. Navigating the TMC often means confronting major political and social backlashes, requiring approaches that minimise the negative repercussions while maximising the short- and long-term benefits. This may mean identifying and acting on windows of opportunity. Experience suggests (for example, in water management in Vietnam: (Nguyen et al. 2012)) that it is important to encourage open enquiry, and to explore different framings and strategies for a given situation, rather than fixing on one approach and applying it regardless of the context.

Third, pursuing these strategies implies that there is a need to **increase capabilities to cope with and learn about the tensions** and their navigation—in fact, to seek to mainstream literacy about transformation (since literacy about mainstreaming is, after all, generally mainstream) and its intent across organisations. Even though transformation is not the primary day-to-day focus of most staff, it helps if everyone is aware of the TMC and appreciates the legitimacy of the part of the institutional arrangements that have the mandate and power to imagine transformative futures. This enables those actors to design mechanisms and pathways towards transformation, whether these are internal or external (e.g. taskforces or royal commissions). People must anticipate the emergence of complex and ambiguous ethical and political dimensions of both the direction and approach of such activities (Eriksen et al. 2015; Scoones 2016; Nightingale et al. 2022), which are likely to be in tension with mainstream business-as-usual. Context-specificity and uncertainty mean that continuous learning and improvement need to be encouraged (e.g. “evolutionary learning”—(Ansell and Gash 2007; van Kerkhoff et al. 2019)). Transformative adaptation also requires specific capabilities and competencies in developing near-term actions that anticipate and catalyse longer-term systemic changes. These can lead to proactive, collective action while navigating uncertainty and the complexities of the evolving risks (Kates et al. 2012; Pelling et al. 2014; Schipper et al. 2021; Meharg 2022), for example, using adaptation pathways (Haasnoot et al. 2024). This requires not only human capabilities but also attention to organisational arrangements. These include policies or procedures that may undermine support for transformation, often unintentionally, such as the expectation of neat “solutions” that fit within mainstream paradigms.

Fourth, this requires the mandate, knowledge and support of upper management and stakeholders for adaptation planning and implementation, requiring **a shift in leadership models and approaches to become more facilitative and systems-based**, rather than formal and hierarchical. These changed models can still be used to manage business-as-usual organisations but are *necessary* to allow leaders to be comfortable with accepting the ambiguity of the TMC and, critically, for them to enable and endorse others to recognise, struggle with and navigate the tensions that arise. Brassey et al. (2021) described this as moving from a “status quo mindset” to an “adaptable learning mindset” and provided pointers for how to nurture such leadership change. In the context of the TMC, leadership must mean more than dictating simple responses to the tensions that “pragmatically” favour one imperative over the other.

Fifth, organisations can deploy a range of extant tools that can help conceptualise and implement adaptation and other systems change initiatives, including addressing mainstreaming and transformation aspirations. However, there is a need to **adapt, develop, test and share tools and strategies for navigating the TMC tensions** in practice. Several existing models can help practitioners conceptualise how mainstreaming and transformation can be simultaneously parts of a larger process. For example, Geels (2019) describes technological transitions as transformative processes requiring multiple layers of societal change, often needing the mainstream sponsoring of “protected niches” where change can be initiated and grow, before being accepted into and transforming the broader societal “landscape”. More specific tools can address some of the tensions: for example, the Cynefin framework (Kurtz and Snowden 2003) emphasises the different entailments of complex and complicated spaces. Pragmatic complexity (Ansell and Geyer 2017) can provide a sound conceptual basis for a world where paradoxes are prevalent. Wardley mapping (Wardley 2020) can help articulate the tensions and strategies where emerging challenges need to work within existing systems and across scales. Similarly, concepts and methods from resilience thinking, transdisciplinary approaches, adaptive governance and systems thinking can all be useful in different contexts.

## Conclusions

Adapting to climate change is becoming increasingly urgent, but this perspective article has identified a conundrum that arises when seeking to balance transformative and mainstreaming approaches to adaptation. The TMC can trigger multiple, persistent tensions, seven of which we have discussed (Table 1); these impede the planning and implementation of mainstreamed transformative adaptation and can be seen as frustrating barriers to action. However, if they are recognised, they can also be navigated constructively. Indeed, this recognition and navigation is necessary to progress adaptation at a level that is appropriate to our contemporary challenges.

This perspective article has identified five strategies to assist with addressing the TMC (Fig. 1), starting with acknowledging the conundrum and reframing the tensions as foreseeable challenges rather than blockages to action. Then, practitioners need to adopt context-specific strategies to navigate the tensions, for which we suggest some generic steps: actively increase the capability of individuals and organisations to address the tensions; shift leadership models to support this navigation; and work to expand the range of tools available to help. We call on researchers to help with these efforts by paying attention to the TMC as a recognisable issue, exploring how the tensions play out and testing what strategies can best navigate the TMC in different contexts. The list of tensions and responses in Fig. 1 is unlikely to be complete: We urge others to share their stories of these and other tensions and the lessons learned in navigating them to re-energise the urgency of scaling up adaptation efforts.
